# Supplementary material for: Serum-deprivation response of ARPE-19 cells; expression patterns relevant to age-related macular degeneration
Source: PLoS One. 2024 Sep 26;19(9):e0293383. doi: 10.1371/journal.pone.0293383 (PMC11426544; doi:10.1371/journal.pone.0293383)
Supplement: S1 Fig — Information and links for the interactive figures that accompany this paper. (DOCX) [file pone.0293383.s004.docx]

**S1Figure. Interactive Figure information.** These seven interactive figures to accompany Peterson et al. PLoS ONE 2024, are hosted on the Tableau Public website ([https://public.tableau.com](https://public.tableau.com/)). The visualizations allow the reader to view the data interactively and to sort and filter the data for greater understanding and insight.

**Interactive Figure 1. Genes included in the gene set analysis; the effect of using multiple gene set database collections.** Interactive figures for exploring the gene set annotations and gene set collections used in this analysis.

[https://public.tableau.com/views/S1FigureforPeterson/DatabasesUsed?:language=en-US&:sid=&:display_count=n&:origin=viz_share_link](https://public.tableau.com/views/S1FigureforPeterson/DatabasesUsed?:language=en-US&:display_count=n&:origin=viz_share_link)

**Interactive Figure 2. Analysis and interpretation using interactive visualizations.** Interactive figure for exploring the data structure and summaries of the analyses. Displays the major graph types used and provides instruction for interaction.

<https://public.tableau.com/views/S2_FigureforPeterson/FigS2_HowtoReadtheGraphs?:language=en-US&:display_count=n&:origin=viz_share_link>

**Interactive Figure 3. EGSEA Analysis and Interpretation of Cholesterol and Lipid Gene Sets.** Interactive figure for exploring the cholesterol and lipid gene sets identified by EGESEA analysis.

<https://public.tableau.com/views/S3Figure/FigureS3?:language=en-US&:display_count=n&:origin=viz_share_link>

**Interactive Figure 4. Differential Expression of Genes Encoding the Extracellular Matrix.** Interactive figure for exploring the gene expression changes associated with the extracellular matrix genes.

<https://public.tableau.com/views/S4Figure_forPeterson_etal/FigureS4?:language=en-US&:display_count=n&:origin=viz_share_link>

**Interactive Figure 5. Cells display signs of senescence at the end of the time series.** Interactive figure of EGSEA results for gene sets involved in cell senescence.

<https://public.tableau.com/views/S5Figure_forPeterson_etal/FigureS5?:language=en-US&:display_count=n&:origin=viz_share_link>

**Interactive Figure 6. Differential Expression of Inflammation, Complement, and Coagulation Gene Sets.** Interactive figure of EGSEA results for inflammation, complement, and coagulation gene sets.

<https://public.tableau.com/views/S6Figure_forPeterson_etal_/FigureS6?:language=en-US&:display_count=n&:origin=viz_share_link>

**Interactive Figure 7. Circadian Function Genes and Unannotated DEGs.** Interactive figure to explore gene sets involved in circadian rhythm, and to examine DEGs without annotations from the major gene set collections used in this analysis.

<https://public.tableau.com/views/S7Figure_forPeterson_etal/FigureS7?:language=en-US&:display_count=n&:origin=viz_share_link>
