# Supplementary material for: Serum-deprivation response of ARPE-19 cells; expression patterns relevant to age-related macular degeneration
Source: PLoS One. 2024 Sep 26;19(9):e0293383. doi: 10.1371/journal.pone.0293383 (PMC11426544; doi:10.1371/journal.pone.0293383)
Supplement: Attachment — Submitted filename: Response to Reviewers.docx [file pone.0293383.s005.docx]

Katherine MacLean Peterson

MSFG, NEI, NIH

January 24, 2024

Dear Editor and Reviewer,

Thank you for your thoughtful and careful review of our manuscript, “Serum-deprivation response of ARPE-19 cells; expression patterns relevant to age-related macular degeneration”.

Here is our response:

1. To our knowledge, the re-submitted manuscript meets PLOS ONE’s style requirements.
2. We note that the grant information you provided in the ‘Funding Information’ and ‘Financial Disclosure’ sections do not match. I apologize for the confusion. Our Funding Information statement should read, “This research was supported by the Intramural Research Program of the NIH, National Eye Institute.”
3. We are unable to open your Supporting Information file [S1_Table.txt ]. Please kindly revise as necessary and re-upload. We have uploaded the new and renumbered supplementary tables. The new S2_Table is a text file that can be opened using a text editor or imported into Excel. We have tested this file using both Windows and Mac-based computers.
4. Please review your reference list to ensure that it is complete and correct. We have reviewed our reference list, and to our knowledge, none of the papers have been retracted.

The Reviewer’s Comments, followed by our response.

The manuscript employed ARPE-19 cells under serum deprivation to model RPE in AMD and analyzed gene expression changes and signaling pathways via RNA sequencing and GSEA analysis. It reported alterations in AMD-associated pathways in serum-deprived ARPE-19 cells.
Major comments:
1. ARPE-19 cells, due to their low expression of RPE signature genes and poor transepithelial electrical resistance scores, do not accurately represent RPE cells. This study utilized cells at 80% confluence, which even lacked the critical RPE polarity. Models such as iPSC-RPE or primary human RPE cells offer more accurate in vitro representations for these analyses. Thus, the limitations of the ARPE-19 cell model significantly undermine the study's relevance to AMD.

In this and previous papers, we have explained that we are not using ARPE-19 cells as a model for mature, differentiated RPE cells. Rather, we are studying a stress response exhibited by these cells, which are derived from mature RPE. We discovered that serum deprivation of ARPE-19 led to increased secretion of EFEMP1/Fibulin 3, a protein deposited at Bruch’s membrane in AMD and in Doyne’s Macular dystrophy. This observation led to experiments to characterize other aspects of the serum-deprivation response. As we have already shown and further characterized in this paper, the cells exhibit a repertoire of responses relevant to AMD. These include upregulation of lipid and cholesterol pathways, differential patterns of cholesterol accumulation, secretion of Fibulin 3 and ApoB, and deposition of calcium in the form of hydroxyapatite (HAP). All these also occur in dry AMD.

We hypothesize that ARPE-19 cells, having gone through all the stages of chromatin development from stem cells to mature RPE, retain some programmed responses, such as the response to serum deprivation, typical to native RPE. In this paper, we have used RNASeq to characterize the response of ARPE-19 cells to serum deprivation more completely. As shown, we confirm the previous observations and extend them to show several other transcriptional responses, including changes in ECM protein expression, that are also similar to AMD. This ARPE-19 model led to our discovery of the induction of amelotin in response to serum deprivation, its essential role in HAP formation, and its similar expression in AMD, where it is associated with the HAP deposits that are now recognized as markers for disease progression. We contend this is a significant finding with major implications in the AMD field.

The reviewer asserts that the ARPE-19 cells do not express RPE markers. As shown in the new Table, S1 Table, they do. However, we repeat that we are not using these cells as a model for fully differentiated, healthy RPE but as a tool for exploring a disease-relevant stress response. RPE cells affected by AMD are no longer healthy, normally functioning RPE. They are under stress and are exhibiting responses that likely contribute to disease progression. Our results have shown that our cell model has many features relevant to AMD, and it has already proved to be a powerful tool for discovery.

S1 Table. Gene expression measures, represented by RNASeq counts, of RPE marker proteins identified by Reyes et al., Nature Communications (2020)11: 1609, during serum deprivation of ARPE-19 cells. The table presents normalized RNASeq counts determined by DESeq for each sample day. The values are mean values from three biological replicates performed in triplicate.

The manuscript could benefit from improvements in readability and structure:
Figures should be presented sequentially as they are referred to in the text. For instance, Figure S5B, which illustrates ECM genes, could be incorporated into Figure S4. Moreover, Figure 4's discussion (lines 291-294) precedes that of Figure 3; hence, it would be logical to introduce Figure 4 earlier in the text to maintain coherence.

The authors appreciate the reviewer’s thoughtful comments about the structure and organization of the ECM results. We have edited this section to address their concerns. First, we have renumbered the figures, changing the IHC image from Figure 3 to Figure 4. Secondly, we have reworked a new Figure 3, a highlight figure of the differentially expressed ECM genes. We believe that the new Figure 3 will aid viewer comprehension and reduce the redundancy of the supplementary figures.

The supplementary material contains a lot of information that requires frequent access. Consider integrating crucial figures into the main text. Duplication of figures in both the main and supplementary sections leads to redundancy.
Explicitly connect figure parts to the corresponding textual statements. For example, after the statement in lines 254-255 regarding triglyceride biosynthesis, directly refer to the specific panel in Figure S3 that supports this claim to guide the reader effectively.

We agree that the supplementary interactive figures are critical to evaluating and understanding our data and wish there were some means to integrate them directly into the paper. The interactivity of the figures allows us to employ the best practices of analyzing and displaying large datasets by providing an overview, zoom and filter capability, and details on demand. Unless or until PLoS incorporates a Tableau server into its platform, we must employ the compromise of the Tableau Public server.

We have edited the text in lines 254-255, noted in the review, and included a specific reference to Figure S3C.

Minor Comments

The claim in lines 262-263 regarding serum deprivation effects on ARPE-19 cells needs direct support from Figure S3, which currently appears to be missing.

We have corrected the citation to SFig 3 in lines 262-263.

In discussing the extracellular matrix, a heatmap illustrating the fold changes of the genes mentioned in the main text, such as MMP2 and TIMP3 would enhance comprehension.
The manuscript does not clearly identify the genes related to fibrotic extracellular matrix within the figures.

The new Figure 3 and references to S4C and S5B identify genes related to fibrotic extracellular matrix.

The abbreviations ADAM and ADAMTS should be spelled out upon first use to ensure clarity for all readers.

We have spelled out the abbreviations for ADAM and ADAMTS at first use.

The statement in line 379 about senescence in the context of AMD cites a study using ARPE-19 cell models, which is insufficient to substantiate the role of senescence in AMD.

We have added additional references to support the hypotheses regarding the role of senescence in AMD.

Immunostaining figures should use arrows to delineate the RPE layer from the choroid, so that the staining signals can be distinguished. In addition, the number of human donor eyes utilized for staining should be disclosed.

We added arrows to the IHC Figure 4 (3 that was). We added the number of human donor eyes utilized to the methods section.

For volcano plot figures and legends:
Axis labels need clarity; if P-value is log-scaled, the figure legend should specify it, for example -log10(P-value).
The reference line labeled "0.05" is inconsistent with a log scale. It should reflect the corresponding -log10(0.05) value if the Y-axis is indeed log-scaled.

We have changed the legends for the volcano plots to aid in comprehension. To ensure that our description is consistent with the PLoS style, we modeled our volcano plot legends on those in Hatzirodos et al. (2015) (<https://journals.plos.org/plosone/article?id=10.1371/journal.pone.0119800>).

In Figure 7, a color scale bar could more efficiently convey expression levels, streamlining the figure.

We agree that a color scale bar would streamline Figure 7. However, the data displayed in Figure 7 is not configured for this type of scale; it is discrete and categorical, with binned values for expression level rather than continuous values.

Sincerely,

Katherine Peterson
